# Updates in the Management of Leg Length Discrepancy: A Systematic Review

**DOI:** 10.7759/cureus.62599

**Published:** 2024-06-18

**Authors:** Mohammed Saad Althobaiti, Lama I Aloraini, Saud Alamri, Omar Khalid Binsaddik, Yousef Mansour Y Alobaysi, Faisal K Alabdulrahman, Omar Awdah Saeed Al shahrani, Raghad Mahdi M Al-Awn, Ghady Ahmad Shafiy

**Affiliations:** 1 Orthopedics, King Abdullah Medical Complex, Jeddah, SAU; 2 Orthopedic Surgery, National Guard Hospital, Al Ahsa, SAU; 3 Medicine, King Saud Bin Abdulaziz University for Health Sciences, Riyadh, SAU; 4 General Practice, Maternity and Children Hospital, Abha, SAU; 5 General Practice, Tawiq Primary Health Center, Riyadh, SAU; 6 Medicine, King Khalid University, Riyadh, SAU; 7 Medicine and Surgery, King Khalid University Hospital, Abha, SAU; 8 Plastic Surgery, Alnoor Specialist Hospital, Mecca, SAU

**Keywords:** gait disturbances, correction, management, musculoskeletal conditions, leg length discrepancy

## Abstract

The aim of this study is to comprehensively investigate the recent literature on the management of leg length discrepancy (LLD). A thorough search of pertinent databases was done in order to find studies that satisfied the requirements for inclusion. A thorough search of PubMed, Web of Science, Scopus, and Science Direct was conducted to find pertinent literature. Rayyan Qatar Computing Research Institute (QRCI, Ar Rayyan, Qatar) was utilized during the whole operation. Eight studies, including a total of 345 patients, were included in our data, and 206 (59.7%) of them were males. Percutaneous epiphysiodesis was the surgical intervention of choice in four studies. LLD can be effectively corrected by temporary and permanent epiphysiodesis. One study reported the incidence of angular deformities following temporary epiphysiodesis. Circumferential periosteal and dual tension-band plating significantly reduced LLD, but reported the incidence of an "over-shoot" in some patients. Bilateral motion control shoes and orthotic insole both were found to improve the patient's gait and trunk symmetry, evidenced by longer and faster steps, reduced ground impact at heel strike, and lower peak plantar pressure in both limbs. Our findings confirm that no inferences about the superiority of a particular management approach for the treatment of LLD can be made. The poor quality of the studies shows that more randomized control trials and prospective studies on the subject are required.

## Introduction and background

Anisomelia, also known as leg length discrepancy (LLD), is a disorder in which there is a noticeable difference in the length of the paired lower extremity limbs. The prevalence of LLD higher than 2 cm is 1/1000 [1]. LLD can be caused by a variety of factors beyond infection, including congenital abnormalities, trauma, tumors, inflammatory conditions, and neuromuscular disorders. Conditions present at birth, injuries, growth plate issues, benign or malignant growths, joint inflammation, and muscle imbalances are all potential contributors to differences in leg length [2]. While small variations in limb length might not have a major influence on daily living activities, those with an LLD of 2 cm or more may suffer from a number of negative consequences [3]. These can include an uneven gait, low back pain (LBP), hip and knee osteoarthritis (OA) risk, changed lumbar spine biomechanics, and a sense of imbalance [4,5].

Near normal post-pubertal lower limb length and equal leg lengths are the goals of managing LLD. Numerous therapeutic approaches have been employed, such as orthotics, surgical procedures, and shoe lifts. Closing the growth plate by the technique of epiphysiodesis is thought to be the best course of action for LLDs are expected to be between 2 and 5 cm [2]. When there is sufficient remaining development potential and the growth plates (physes) are still open, this therapy approach is appropriate. Epiphysiodesis comes in two main varieties: transient and permanent. In order to treat LLD, a reversible technique called temporary epiphysiodesis slows down the development plate temporarily [6]. Permanent epiphysiodesis refers to an irreversible intervention, where the growth plate is closed permanently, and the goal is to achieve leg-length equality while minimizing the risk of complications associated with more invasive surgical procedures, such as limb lengthening [7]. The most used techniques for permanent epiphysiodesis are percutaneous epiphysiodesis (PE) with drills/curettes and Phemister/modified Phemister technique [8,9].

The degree of the discrepancy and the intensity of the symptoms have always been taken into account when managing LLD [10]. One of the most popular therapies for LLD is a shoe lift, which has several benefits such as being non-invasive, affordable, simple to apply, and quickly removable if desired [11]. For patients suffering from LBP, hip OA, or knee OA, shoe lifts can help correct LLD in order to reduce pain and enhance functional results [12]. The aim of this study is to provide a comprehensive overview of the current management strategies for LLD, focusing on the latest updates and advancements in treatment options.

## Review

Methods

This systematic review used the criteria outlined in the Preferred Reporting Items for Systematic Reviews and Meta-Analyses (PRISMA) [13]. An electronic systematic search of electronic databases including PubMed, Web of Science, SCOPUS, and Science Direct was conducted to identify relevant studies published in English. The search strategy included keywords related to the management of LLD. The following keywords were used: “Leg length discrepancy,” “Correction,” and “Management.” Two reviewers independently screened the search results, selected appropriate studies, extracted data, and assessed the quality assessment of the included research using appropriate evaluation tools.

The eligibility criteria for inclusion in this study encompass several key points. Firstly, studies must focus on the management of LLD. Additionally, only studies involving patients with LLD as the primary condition, without any other associated factors, were considered. The timeframe for eligible studies is between 2022 and 2024, and they must be available in English. Furthermore, the studies should involve human subjects across all age groups and can take the form of randomized controlled trials, observational studies, cross-sectional studies, case-control studies, cohort studies, or case series. On the other hand, exclusion criteria specify that studies unrelated to LLD management will be excluded. Additionally, animal studies, in vitro studies, and review articles lacking original data were considered. Studies with insufficient or unclear information regarding surgical management and those offering weak evidence, such as case studies or expert opinions without primary data, were also excluded from the analysis.

Data Extraction

To guarantee correctness, the search results were validated using Rayyan (rayyan.qcri.org) [14]. Titles and abstracts found in the search results were evaluated for relevancy using both inclusion and exclusion criteria. Papers that satisfied the inclusion requirements were carefully examined by the study team. A discussion was used to settle any disagreements. Key study information, including titles, authors, publication year, study location, participant characteristics, gender distribution, intervention management, management methods, follow-up duration, and main outcomes, was recorded using a preset data extraction template. An independent document was made to assess the potential for bias.

Data Synthesis Strategy

To offer a qualitative assessment of the research findings and components, summary tables were created using data taken from pertinent studies. Following the collection of data for the systematic review, the best strategy for making use of the data from the included studies was identified.

Risk of Bias Assessment

To assess the quality of the study, the Joanna Briggs Institute (JBI) [15] critical assessment criteria for studies reporting prevalence data were used. This tool consists of nine questions. Positive answers receive a score of 1, while negative, unclear, or irrelevant answers receive a score of 0. Ratings of less than 4, five to seven, and more than eight were categorized as low, moderate, and outstanding quality, in that order. Scholars evaluated the quality of the work in their own right, and disagreements were settled by discussion.

Results

Search Results

**Figure 1:**
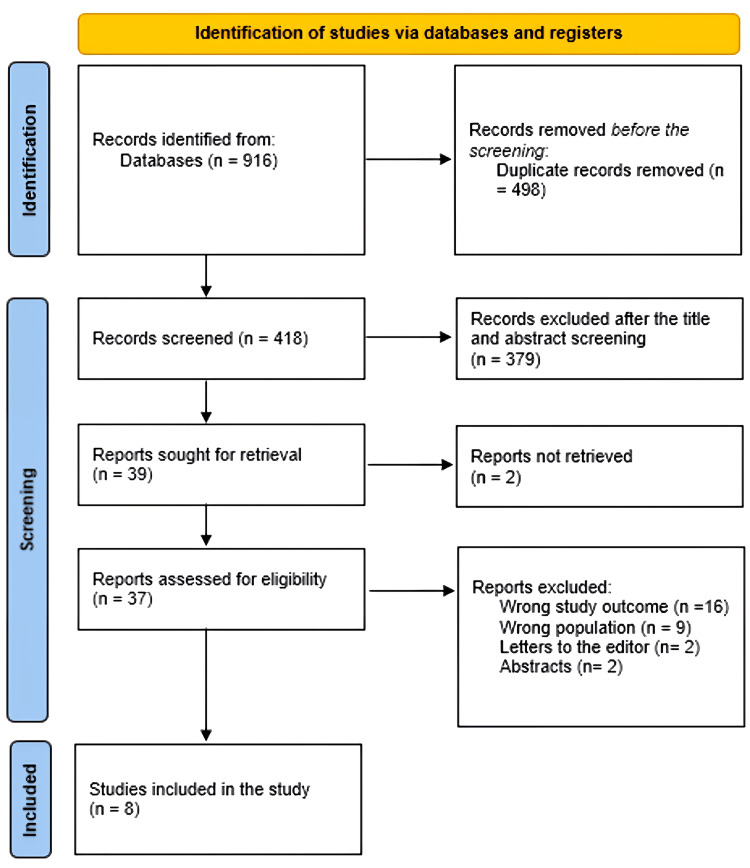
Study decision is summed up in a PRISMA diagram. Preferred Reporting Items for Systematic Reviews and Meta-Analyses (PRISMA)

After 498 duplicates were removed, a total of 916 study papers were found through a systematic search. After 418 studies had their titles and abstracts evaluated, 379 papers were discarded. Merely two articles were not located out of the 39 reports that were required to be retrieved. Thirty-seven papers were screened for full-text assessment; 19 were rejected because the study results were wrong, nine because the population type was inaccurate, two articles were editor's letters, and two were abstracts. Eight research publications in this systematic review satisfied the requirements for eligibility. An overview of the procedure used to choose the research is illustrated in Figure 1. 

Sociodemographic Features of the Comprised Studies

The research publications' sociodemographic information is displayed in Table 1. Eight studies including a total of 345 patients - 206 (59.7%) of them were males - were included in our data. All of the included studies were retrospective in nature [16-23], five were cohorts [16,17,19,20,23], two were case series [18, 21], and one was cross-sectional [22]. Two studies were conducted in the UK [18,19], one in Norway [16], one in Russia [16], one in Poland [20], one in Turkey [21], one in Taiwan [22], and one in China [23]. 

**Table 1:** The sociodemographic attributes of the participating populations.

Study	Study design	Country	Participants	Mean age	Males (%)
Weinmayer et al., 2022 [16]	Retrospective cohort	Norway	88	13.2	49 (55.7%)
Petrova et al., 2022 [17]	Retrospective cohort	Russia	94	2 to 14	56 (59.6%)
Chatterton et al., 2023 [18]	Retrospective case series	UK	18	5.3	11 (61.1%)
Tolk et al., 2022 [19]	Retrospective cohort	UK	42	12.1 ± 1.7	25 (73.5%)
Starobrat et al., 2024 [20]	Retrospective cohort	Poland	60	13.2	36 (60%)
Demirel et al., 2022 [21]	Retrospective case series	Turkey	11	6 to 11	7 (63.6%)
Jankaew et al., 2023 [22]	Retrospective cross-sectional	Taiwan	20	21.95 ± 2.2	16 (80%)
Shi et al., 2022 [23]	Retrospective cohort	China	12	9.0 ± 2.3	6 (50%)

Clinical Outcomes

The clinical features are displayed in Table 2. Out of the eight studies, six discussed correction surgical interventions [16-21] and only two included symptomatic treatments for gait disturbances [22,23]. Percutaneous epiphysiodesis was the surgical intervention of choice in four studies [16,17,20,21]. Two studies reported using eight-shaped plates, which showed the most effective results [17,21]. LLD can be effectively corrected by temporary epiphysiodesis [16,17,20,21]. One study reported the incidence of angular deformities following temporary epiphysiodesis [16].

**Table 2:** Clinical features and results of the included research. *NM (Not Mentioned)

Study	Intervention	Management method	Follow-up duration (months)	Main outcomes	JBI
Weinmayer et al., 2022 [16]	Correction	Percutaneous epiphysiodesis	12	Following percutaneous epiphysiodesis, they discovered a significant incidence of secondary angular abnormalities, and the degree of the deformities was connected with the degree of growth that was still present at the time of operation.	High
Petrova et al., 2022 [17]	Correction	Temporary epiphysiodesis using 8-shaped plates	NM	In pediatric patients, LLD can be effectively corrected by temporary epiphysiodesis using 8-shaped plates. When fixing the thigh length disparity as opposed to the lower LLD, it is more effective.	Moderate
Chatterton et al., 2023 [18]	Correction	Circumferential periosteal release	63.1 ± 33.9	Actual and percentage LLD is significantly reduced by circumferential periosteal release. But it can happen at any time, and some patients with a slight disparity might "over-shoot" a little bit.	Moderate
Tolk et al., 2022 [19]	Correction	Dual tension-band plates	6	Dual tension-band plating can be used to significantly reduce LLD. Only the proximal tibia showed a change in intra-articular morphology; the distal femur did not show this same change.	Moderate
Starobrat et al., 2024 [20]	Correction	Temporary epiphysiodesis	42	Over the course of treatment, epiphysiodesis changes the proximal tibial articular surface.	High
Demirel et al., 2022 [21]	Correction	Temporary hemiepiphysiodesis using 8-shaped plates	62	Temporary hemiepiphysiodesis utilizing the eight-plate appears to be a successful treatment with low complication rates for the management of children with LLD of 2-4 cm.	Moderate
Jankaew et al., 2023 [22]	Symptomatic management	Bilateral motion control shoes	6	Bilateral motion control shoes with adjustable outsoles can reduce ground impact at heel strike and enhance trunk symmetry.	Moderate
Shi et al., 2022 [23]	Symptomatic management	Orthotic insole	6	When using an orthotic insole, children with mild LLD demonstrated an immediate improvement in their gait, as evidenced by longer and faster steps and lower peak plantar pressure in both limbs.	Moderate

Circumferential periosteal release was used in one study [18] and reported that LLD was significantly reduced, but reported the incidence of an "over-shoot" in some patients [18].

Another study reported that dual tension-band plating can be used to significantly reduce LLD. Only the proximal tibia showed a change in intra-articular morphology; the distal femur did not show this same change [19].

Bilateral motion control shoes [22] and orthotic insole [23] both were found to improve the patient's gait and trunk symmetry evidenced by longer and faster steps, reduced ground impact at heel strike, and lower peak plantar pressure in both limbs.

Discussion

To our knowledge, this is the first systematic review to investigate the various management approaches to LLD in a pediatric population. In this review, percutaneous epiphysiodesis was the surgical intervention of choice in four studies [16,17,20,21]. Two studies reported using eight-shaped plates, which showed the most effective results [17,21]. LLD can be effectively corrected by temporary epiphysiodesis [16,17,20,21]. One study reported the incidence of angular deformities following temporary epiphysiodesis [16]. Tirta et al. reported that over 50 years have passed since the first permanent epiphysiodesis was carried out. The current state of the relatively small surgery of permanent epiphysiodesis is interesting, nonetheless, given the availability of innovative LLD therapy alternatives such as motorized lengthening nails. A pooled analysis of the available literature revealed a 17.5% complication rate and a 73.7% success rate for permanent epiphysiodesis operations in this systematic review [24].

Closing the growth plate by the technique of epiphysiodesis is thought to be the best course of action for kids whose LLDs are expected to be between 2 and 5 cm when they reach adulthood [2]. When there is sufficient remaining development potential and the growth plates (physes) are still open, this therapy approach is appropriate. Epiphysiodesis comes in two main varieties: transient and permanent. In order to treat LLD, a reversible technique called temporary epiphysiodesis slows down the development plate temporarily [6]. By permanently closing the growth plate, permanent epiphysiodesis is an irreversible operation that aims to achieve leg length equality while reducing the risk of complications associated with more invasive surgical treatments, such as limb lengthening [7]. Percutaneous epiphysiodesis with drills or curettes and Phemister/modified Phemister technique are the most commonly utilized methods for permanent epiphysiodesis [8].

An infrequently documented method of pediatric leg lengthening, circumferential periosteal release corrects the short leg rather than confining the longer limb and avoids many of the difficulties associated with intramedullary devices or frames. Longitudinal stripping was originally documented clinically in 1975 as a treatment for individuals with limb length disparities following poliomyelitis. Wilde and Baker provided the technique's first detailed description in 1987. A strip of periosteum from the distal femur, tibia, and fibula's metaphysis is excised circumferentially during the surgery [25]. It is still unknown how exactly this method causes the limb to expand. According to the vascular model, sectioning the periosteum causes a localized hyperaemic reaction [26], which promotes bone development. This effect is also observed in the overgrowth that results from pediatric femur shaft fractures [27]. Another concept that involves the periosteum acting as a mechanical constraint on the physis is mechanical in nature [28]. Additionally, periosteal sectioning has been demonstrated in animal models to alter chondrocyte activity within the physis [29].

Another study in this review reported that dual tension-band plating can be used to significantly reduce LLD. Only the proximal tibia showed a change in intra-articular morphology; the distal femur did not show this same change [19]. For longitudinally guided growth, dual tension-band plates are employed as a temporary epiphysiodesis technique [29-31]. While the growth-limiting effect is said to be slower and less predictable than with definitive surgical physeal ablation, a growth-modulating effect is nevertheless anticipated [29,31,32]. There is no documented difference in the correction rate or residual LLD when compared to alternative temporary epiphysiodesis methods (such as Blount staples) [33]. Concerns about the reversibility of epiphysiodesis have been raised when hard staples are used in LLD repair. The inflexible physeal compression may cause a total growth stop [34,35]. With a possible lower chance of total physeal arrest, LLD repair with tension-band plates seeks to decelerate growth rather than completely stop it [34,36].

In the sagittal and frontal planes of the stance and swing phases, the common compensating mechanism is the kinematic modification of the trunk, pelvis, and lower limbs. Many compensatory strategies are used by LLD participants: for the long leg, these include downward pelvic tilt, exacerbated hip and knee flexion, increased hip abduction, and increased ankle dorsiflexion angle; for the short leg, they include upward pelvic tilt, decreased hip and knee flexion, reduced hip adduction, and increased ankle plantar flexion angle [37,38]. Regarding the symptomatic management reported in the present review, bilateral motion control shoes [22] and orthotic insole [23] both were reported to improve the patient's gait and trunk symmetry evidenced by longer and faster steps, reduced ground impact at heel strike, and lower peak plantar pressure in both limbs.

Limitations

This review has several limitations. First, it lacks the quantitative analysis due to its qualitative nature. Second, we were limited to a timeline of publications as we did not specify a management modality but included all the updates reported in managing LLD. Third, most of the included studies did not mention the degree of LLD, which may bias our findings as we did not conduct subgroup analysis for the results. Lastly, the retrospective nature of all the included studies is a source of bias for this review, as most studies did not mention the underlying etiology of LLD in the participants and did not mention the indication of using a certain modality for the management.

## Conclusions

Our findings confirm that no inferences about the superiority of a particular management approach for the treatment of LLD can be made. The poor quality of the studies shows that more randomized control trials and prospective studies on the subject are required. By addressing the limitations identified in our review, future studies can provide more robust evidence to guide clinical practice and improve outcomes for individuals with LLD.
